# Effect of dilution of stool soluble component on growth and development of *Strongyloides stercoralis*

**DOI:** 10.1038/srep10749

**Published:** 2015-06-02

**Authors:** Witthaya Anamnart, Pewpan Maleewong Intapan, Attarat Pattanawongsa, Pennapa Chamavit, Supreecha Kaewsawat, Wanchai Maleewong

**Affiliations:** 1 Department of Medical Technology, School of Allied Health Sciences and Public Health, Walailak University, Thasala, Nakhon Si Thammarat 80161, Thailand; 2 Department of Parasitology and Research and Diagnostic Center for Emerging Infectious Diseases, Faculty of Medicine, Khon Kaen University, Khon Kaen 40002, Thailand; 3 School of Pharmacy, Walailak University, Thasala, Nakhon Si Thammarat 80161, Thailand; 4 Faculty of Medical Technology, Huachiew Chalermprakiet University, Samut Prakan 10540, Thailand; 5 School of Allied Health Sciences and Public Health, Walailak University, Thasala, Nakhon Si Thammarat 80161, Thailand

## Abstract

Dispersion or dilution of stool by water from heavy rainfall may affect *Strongyloides stercoralis* free-living development producing infective filariform larvae (FL). This study examined effect of water dilution of stool on survival of *S. stercoralis* free-living development. One g of stool was prepared in water so that its soluble component was diluted sequentially from 1:2 to 1:480. Three dishes were used to compare FL production in three culture conditions: stool suspension, stool sediment deposited in soil, and isolated rhabditiform larvae (RhL) deposited in soil. The fourth dish was for developmental observation of RhL into free-living stages. Numerous FL were generated from undiluted or 1:2 diluted stool and stool sediment placed on soil. However, starting from dilution 1:5, FL production continuously decreased in both stool suspensions and stool sediments placed on soil. RhL isolated from stool dilutions placed on soil gave rise to few FL. Worm mating were seen at 24-30 hours in dilutions 1:20-1:120 only. Highest numbers of FL from indirect free-living cycle were 1/3 of those from control. FL production decreased as stool dilution increased, and reached zero production at 1:160 dilution. Rainfall may disperse or dilute stool so that nutritional supplement for *S. stercoralis* free-living development is insufficient.

*Strongyloides stercoralis*, unlike the other helminths parasitizing humans, is able to reproduce outside the host^1^. This indirect development or free-living generation in fecal-contaminated soil promotes maintenance of infection in endemic areas and in fact contributes to difficulty in control of transmission. It is estimated that 30–100 million people are infected worldwide^2^.

Strongyloidiasis is found mainly in tropical and subtropical countries. In Thailand the overall prevalence is about 23.7%^3^, but varies from one place to another. In recent studies, some communities in the south had 1.8% to 10% prevalence^4^,^5^, which appears to be lower than those reported from other parts of Thailand, e.g. 15.9% in the north^6^, 23.5% in the northeast^7^, and 30.5% in central Thailand around Bangkok^8^. Environmental factors, particularly rainfall, may contribute to such variation because southern Thailand has a long rainy season – 10 months compared with a 4-month-long period in other parts of the country. Rainfall may interrupt growth and development of *S. stercoralis* rhabditiform larvae (RhL) by dispersion of stool mass deposited on the soil. In this way, RhL are dispersed and may lack nutrient supply available from the fecal environment. Consequently, free-living development is retarded.

A preliminary observation revealed that some RhL died when placed in diluted stool suspension, while some survived but gave rise to stunted free-living adult worms. We thus aimed to study the growth and development of *S. stercoralis* RhL in diluted stool or in soil.

## Results

### Effect of water dilution of soluble stool component on worm development

Table 1 shows FL count after 120 h as a yield from various dilutions. Undiluted stool suspension and stool diluted 1:2 gave more than 3,000 FL. However, FL production was dramatically reduced at dilutions of 1:5 or more. Fecal sediment from 1:5 and 1:10 suspensions exposed to the experimentally diluted fecal environment for 3 h was able to generate about 3,000 FL when added to soil. After that, the more diluted the environment, the greater the decrease in FL production. Isolated RhL from all dilutions, on the contrary, failed to generate FL when placed in soil, and gave only 0–2 FL per plate.

### Observation of free-living worm development

Table 2 summarizes adult worm and FL counts after incubation of 1 g of stool in various dilution environments at intervals up to 120 h. Yields of adult worms and FL from the indirect cycle continuously decreased as the dilution increased, while FL generated from the direct cycle remained constant up to 1:320 (Table 2). Mating was observed in dilutions of 1:20 to 1:120. At 1:160 dilution, no mating pairs could be seen. Similarly, at 120 h no FL were found at 1:160 dilution and greater. Morphologically, a few adult worms in dishes containing 1:80–1:160 diluted stool appeared stunted, and sexes were hardly differentiated. These worms died within a few days. In addition, FL generated from both direct and indirect cycles in every dilution could survive for 240 h prior to death.

## Discussion

Rainfall disperses soil-transmitted helminth eggs and helps in spreading the infection^9^. This may not be the case for *S. stercoralis*, however, as RhL need to grow into adult males and females, mate, and produce progeny which finally develop into infective FL. Our unpublished study conducted in Moklan subdistrict of Thasala district, Nakhon Si Thammarat province, southern Thailand, revealed that the prevalence of hookworm (*Necator americanus*) infection was 52% while that of strongyloidiasis was only 13%. In San Pa Tong district, Chiang Mai province in northern Thailand, however, prevalence was found to be as high as 38.8%^10^. It is possible that, in wet areas, rainwater may dilute or disperse stool so that RhL are deprived of nutrition and hence are unable to undergo normal growth and development. This assumption is supported by the present findings that FL production decreased when the stool soluble component was diluted in water at 1:20 or more. Sediment prepared by centrifugation of 1:20 stool suspension yielded a fourfold decrease and 1:40–1:320 produced a tenfold decrease in FL production when compared with undiluted and 1:2–1:10 diluted feces (Table 1). It is possible that essential soluble nutrients in feces are washed out. Supporting evidence came from the observation that RhL which were isolated from feces and placed in soil yielded very few FL (Table 1). In another experiment where development of isolated adult worms was followed, the number of adult worms and FL decreased as dilutions increased (Table 2). These results are in accordance with other study findings that most RhL of *S. ransomi* developed into FL rather than free-living adult worms when cultured in washed fecal sediment^11^. Premvati^12^ showed that the absence of food was one of several factors which exerted an adverse effect directly on RhL of *S. fuelleborni.*

## Methods

### Ethical approval

Patients provided written informed consent to participate in the study. This study and the consent procedure was approved by the Ethics Committee on Human Rights Related to Research Involving Human Subjects, Walailak University (HE no.10/064). The methods were carried out in accordance with the approved guidelines.

### Stool samples

Stool samples were selected based on the following criteria: (a) agar plate culture yielded a large number of filariform larvae of *S. stercoralis*; (b) a direct fecal smear revealed at least 1 motile rhabditiform larva per smear; (c) a modified formalin-ether concentration technique showed at least 40 rhabditiform larvae per gram of stool; and (d) no parasites other than *Strongyloides* larvae were present. Thirty chronic strongyloidiasis patients in Thasala District of Nakhon Si Thammarat, southern Thailand, were included. All stools were subjected to the experiment within 2 h after defecation. Patients had not been treated with any anthelmintic drugs prior to fecal collection. Each subject was asked to collect the entire amount of stool in a 100 ml plastic container.

### Soil

Soil samples were taken from areas near the laboratory for convenience. Using a clean spade, the soil was put in plastic bags and then brought to the laboratory. The soil was tested before use by placing a portion with a diameter of 18 mm and height of about 2–3 mm in the center of a 35 mm dish. Water was added to the dish and left for 30 min at room temperature. The dish was placed under a dissecting microscope and emerging nematode larvae in the water were observed at intervals. Only a few nematode larvae were observed and identified as being neither *Strongyloides* nor hookworm larvae.

### Comparative growth and development of *S. stercoralis* rhabditiform larvae

Room temperature during the experiment was 26–31 °C and relative humidity was 70–85%. Two dishes served as controls: one containing soil, the other the plain surface of a 35 mm dish. One g of stool was placed in each dish.

Eleven stool suspensions were prepared in disposable Petri dishes; each dish contained 1 g of stool in a different volume of water to achieve the required dilutions. Dilutions of 1:2 and 1:5 were prepared in 35 mm dishes by placing 1 g of stool in each dish, adding 1 and 4 ml water, respectively, and then mixing. For the 1:10 dilution, 1 g of stool was mixed with 10 ml water in a 90 mm dish. For dilutions 1:20, 1:40, 1:60, 1:80, 1:120 and 1:160, 1 g of stool was mixed with the corresponding volume of water in a 150 mm dish, then left to settle down for 20 min. The top liquid part was subsequently removed so that each dish still contained sediment equivalent to 1 g of stool; the soluble stool component was then sequentially diluted. To produce dilutions of 1:320 and 1:480, dishes were prepared as for 1:160 and 1:120, but replacement of supernatant fluid with water was done once and twice, respectively. A standard 150 mm dish containing 20 ml water served for a 2 mm depth mark.

Four dishes were prepared for each dilution ranging from 1:20–1:480. Dishes were left standing at room temperature to allow RhL to be exposed to the experimentally diluted fecal environment for 3 h. The content of the first dish was transferred to a tube and centrifuged at 700 × *g* for 5 min; the sediment was then transferred to a 35 mm dish containing soil. The second dish was examined under a dissecting microscope and RhL were isolated into a tube using a Pasteur pipette. The larval suspension was adjusted to a final volume of 0.2 ml with corresponding larva-free stool suspension and then transferred onto the soil in a 35 mm dish. The remaining larva-free stool suspension was kept for daily use to moisten the soil. The third dish was left as is. The fourth dish was subjected to observation of adult worm survival, as described below. After 120 h at room temperature, 5 ml of water was added to soil dishes, allowing FL to migrate into the water. FL were collected and killed with formalin, then counted. For other dishes which did not contain soil, formalin solution was added and FL were counted.

Since RhL could not be isolated from undiluted stool or 1:2 and 1:5 stool suspensions, the experiment with isolated RhL was not performed on these dilutions.

### Effect of water-diluted stool on survival of free-living stages of *Strongyloides stercoralis*

The fourth dish was left standing for 18 h to allow development of RhL into adult worms. All adult worms were isolated and transferred to a 35 mm dish. Worm-free suspension was added to the dish so that the level of suspension was about 2 mm. Dishes were observed under a dissecting microscope at intervals of 24–30, 36, 48, 72, 96, 120 and 240 h. The observation included male and female adult worm count and their mating between 24–30 h, FL count (direct cycle), eggs and RhL at 36 h, and FL count (indirect cycle) at 120 h. Additionally, adult worm viability was followed for up to 120 h, and FL behavior was followed for up to 240 h. The original dish was kept for FL count (direct cycle) at 36 h. An agar plate culture served as a normal control.

### Agar plate culture (APC)

APC was performed as described previously^13^. Briefly, 1 g of each fresh stool sample or stool pellet remaining after centrifugation of stool suspension and kept at various time intervals was placed at the center of a nutrient agar plate and incubated at RT for 5 days (120 h). Worm motility tracks, larvae and free-living adult worms were monitored by stereomicroscope at 1, 24, 48, 72, 96 and 120 h. Ten ml of 10% formalin was added to the agar surface of each dish; worms were then collected for species identification using a compound microscope (× 40), and the number of FL counted.

### Statistical analysis

Descriptive statistics, including mean, standard deviation (SD) and range, were generated using Microsoft Office Excel 2007.

## Additional Information

**How to cite this article**: Anamnart, W. *et al.* Effect of dilution of stool soluble component on growth and development of *Strongyloides stercoralis*. *Sci. Rep.* **5**, 10749; doi: 10.1038/srep10749 (2015).

## Figures and Tables

**Table 1:** Comparative growth and development of *S. stercoralis* rhabditiform larvae from 1 g stool in various dilution conditions in three experimental cultures (*n* = 30).

**Dilutions**	**No. of FL after 120** **h cultivation**^a^			
	**Stool suspension in water**	**Stool sediment added to soil**	**RhL added to soil**^b^	***P***^f^
Undiluted	3,098.0 ±2,301.6^c^ (560–7,900)	3,301.7 ±2,301.7 (720–8,100)	ND^d^	0.733
1:2	3,087.7 ±2,272.1(600–7,700)	3,195.7 ±2,276.3 (650–7,800)	ND	0.855
1:5	0	3,194.3 ±2,319.3(680–7,850)	ND	<0.001
1:10	458.3 ±736.4^e^ (0–2,480)	3,160.7 ±2,300.0(700–7,900)	2.0 ±1.5(0–5)	<0.001
1:20	1,041.7 ±803.8 (70–2,800)	809 ±685.7(60–2,400)	0	<0.001
1:40	932.0 ±723.1(70–2,500)	342.2 ±327.5(20–1,020)	0	0.001
1:60	527.2 ±377.1(82–1,100)	317.0 ±291.4(20–950)	0	<0.001
1:80	445.2 ±307.1(80–900)	276.2 ±264.6(20–800)	0	<0.001
1:120	266.3 ±194.0(45–650)	239.5 ±231.1(15–750)	0	<0.001
1:160	5.1 ±1.8(2–8)	180.2 ±176.9 (10–600)	0	<0.001
1:320	5.3 ±2.2(2–10)	1.4 ±0.8(0–3)	0	<0.001
1:480	0	0	0	1.000

^a^Mean ±SD (range).

^b^No. of RhL ranged from 20–80 RhL in all dilutions.

^c^1 g stool placed on 35 mm dish surface.

^d^ND, not done.

^e^Positive in 10 out of 30 samples.

^f^Non-parametric Kruskal–Wallis was analyzed.

**Table 2:** Growth and development of *S. stercoralis* rhabditiform larvae in various stool dilution conditions (n = 30).

**Dilutions**	**No. of free-living adult worms and filariform larvae (FL) of** ***S. stercoralis***^a^			
	**Female (at 24–30** **h)**	**Male(at 24–30** **h)**	**FL from direct cycle (at 36** **h)**	**FL from indirect cycle(at 120** **h)**
Undiluted	ND^b^	ND	ND	3,293.7 ±2,365.9 (630–8,200)^c^
1:2	ND	ND	ND	ND
1:5	ND	ND	ND	ND
1:10	ND	ND	ND	ND
1:20	27.9 ±19.6 (3–70)	14.4 ±9.7 (1–35)	6.9 ±2.7 (3–12)	1,030.0 ±834.1 (80–3,000)
1:40	25.8 ±18.5 (3–66)	12.4 ±8.5 (2–32)	7.4 ±2.9 (3–12)	1,002.3 ±789.8 (70–2,800)
1:60	17.9 ±11.5 (2–42)	10.1 ±6.3 (1–22)	6.4 ±3.5 (2–15)	517.3 ±369.3 (80–1,100)
1:80	13.2 ±8.7 (0–32)	7.4 ±4.5 (2–16)	5.0 ±2.5 (1–10)	398.7 ±314.2 (0–820)
1:120	9.1 ±5.5 (1–20)	5.4 ±3.5 (0–14)	5.0 ±2.0 (3–9)	209.0 ±168.1 (0–600)
1:160	4.5 ±5.0 (0–16)	2.1 ±1.7 (0–6)	5.3 ±2.1 (2–8)	0
1:320	0	0	5.5 ±2.3 (2–10)	0
1:480	0	0	0	0

^a^Mean ±SD (range).

^b^ND, not done.

^c^Result from APC using 1 g stool.
